# The influence of different sources of anticipated instrumental support on depressive symptoms in older adults

**DOI:** 10.3389/fpubh.2024.1278901

**Published:** 2024-01-30

**Authors:** Duanduan Fu, Fang Wang, Baizhi Gao, Qin Bai, Guilin Liu, Jinghui Zhu

**Affiliations:** School of Public Health and Management, Wenzhou Medical University, Wenzhou, China

**Keywords:** anticipated instrumental support, intergenerational support, depressive symptoms, older adults, China Health and Retirement Longitudinal Study

## Abstract

**Objective:**

This study investigated how anticipated instrumental support sources and intergenerational support influence depressive symptoms in older Chinese adults.

**Methods:**

We employed binary logistic regression on data from 7,117 adults aged ≥60 in the 2018 China Health and Retirement Longitudinal Study, controlling for gender, marital status, and self-rated health.

**Results:**

38.89% of respondents exhibited depressive symptoms. Anticipated support from spouse and children, spouse only, children only, or other sources showed 52, 25, 46, and 40% lower odds of depression, respectively, compared with no anticipated support. Those providing financial support had 36% higher odds of depression than those without exchanges. However, those receiving financial support, receiving instrumental support, and receiving and providing financial and emotional support had 19, 14, 23, and 24% lower odds of depression.

**Conclusion:**

Different anticipated instrumental support sources and intergenerational support influenced depression odds in older adults, suggesting potential benefits in promoting such support systems.

## Introduction

Depression is a significant mental health concern that is prevalent among older populations worldwide. The World Health Organization has indicated that, globally, 15% of older individuals experience some form of mental illness, with depression notably prevalent (1). Particularly in low- and middle-income nations, the magnitude of this issue is pronounced. For instance, depression rates soared to 34.4 and 36.9% among the older populations of India and Bangladesh, respectively (2, 3); far higher than the world average. China also experiences heightened prevalence, with studies noting that 20% of its older population grapples with depression (4). Distressingly, this rate escalates to 40.7% when considering older individuals in rural areas (5). The implications of depression in later life stages are profound: it can induce appetite and weight changes, disruptions in sleep patterns, and feelings of diminished self-worth or undue guilt (6). These manifestations can further compound risks of conditions such as obesity, diabetes, and even severe outcomes such as suicide, disability, or mortality (7–10). Thus, addressing depression among older individuals is an imperative public health priority.

As China’s population ages, the demand for caregiving services for older adults also increases (11). However, caregiving by relatives is being weakened due to factors such as changes in family structure, rapid urbanization, and increased labor mobility (12, 13). Research has found that over 30% of older adult’s caregiving needs are not being met, and 41.67% of older adults have anticipated instrumental needs (14). In addition, as people age, they might encounter various health challenges. For example, frailty, urinary incontinence and an increased risk of falling, etc. (15). In such circumstances, receiving timely instrumental support becomes crucial, as without it the health of older adults may be seriously threatened. Hence, the assurance of anticipating instrumental support, or “anticipated instrumental support,” is significant for them.

Chinese families have always attached great importance to the relationship between upbringing and support, and the filial piety culture contained in it is an excellent culture that China has always inherited. And this relationship mainly manifests as intergenerational support and anticipated instrumental support for older adults (16). Intergenerational support is considered a factor related to depressive symptoms in older adults, and many studies have been conducted in-depth studies from different content and directions of intergenerational support (17–20). These studies focus on the impact of actual support received or provided by older adults on their depression. Anticipated support, the subjective perception of older adults toward future events, may buffer external stress on mental health (21). Studies has found that anticipated support can bring a sense of security to older adults and is negatively associated with depression (22–24). Among the various aspects of anticipated support, older adults anticipated receiving instrumental support from their adult children (25). It can be observed that both intergenerational support and anticipated support may be important influencing factors for depression in older adults. However, there is limited research that simultaneously explores the impact of these two factors on depression in older adults. Therefore, this study aims to investigate the simultaneous impact of intergenerational support and anticipated instrumental support on depressive symptoms in older adults.

Research on the correlation between anticipated instrumental support and depression in older adults has predominantly focused on two areas. The first considers how anticipated support modulates depressive symptoms. For instance, studies leveraging data from the 2011 and 2013 waves of the China Health and Retirement Longitudinal Study (CHARLS) indicate that anticipated instrumental support is linked to a decreased depression risk among older adults (26). In contrast, other research has found that anticipated instrumental support could inadvertently harm certain older adults. For some older adults individuals, receiving such support means a decrease in their physical function and self-care ability, which can induce feelings of inferiority and burden (27). Krause compared the implications of both received intergenerational support and anticipated support on depression, highlighting their distinct and diverse effects on the mental health of older individuals (21). Dong et al. found that the association between anticipated instrumental support and depressive symptoms is influenced by the balance between expected and received instrumental support. Older adults who received greater instrumental support than they expected were more likely to have a lower risk of depressive symptoms, while those with greater instrumental support expectations than actual receipt were more likely to have a higher risk of depressive symptoms (28).

The second focal area concerns the influence of various sources of anticipated support on depressive symptoms in older individuals. Cheng (29) comparative study between Chinese and American older adults, using data from the 2010 and 2012 waves of the Health and Retirement Survey in the United States and the 2011 and 2013 waves of CHARLS in China, discovered that, in China, anticipated instrumental support from children was a more vital protective factor against depression than support from other sources (29). This contrast was not evident in the U.S. data. These disparities are likely rooted in cultural beliefs and systemic paradigms. Within the Chinese cultural framework, there is a deeply ingrained ethos of children serving as primary caregivers during their parents’ later years, exemplifying the revered tenet of filial piety. Such caregiving, apart from satisfying emotional yearnings (30, 31), addresses tangible needs, especially against the backdrop of China’s limited institutional older adults care and social welfare infrastructure (32, 33). Given this context, the distinct sources of anticipated instrumental support in China must be dissected. Understanding their varied impact on depression can unveil the interplay between traditional family expectations, present-day social systems, and the well-being of older adults.

In summary, there are several limitations to current studies. Firstly, most studies tend to analyze the relationship between intergenerational support or anticipated instrumental support and depressive symptoms in older adults separately, with few studies integrating intergenerational support and anticipated instrumental support to discern their collective implications for depression. Given that both domains distinctly influence mental well-being in older adults, an exclusive focus on either facet could inadvertently introduce analytical biases (26, 34). Secondly, the research that concentrates on older populations in China largely draws upon survey data from a decade past. This temporal distance is pertinent, considering the substantial shifts that have transpired in China’s social welfare landscape, elder care paradigms, and familial configurations (35, 36). Thus, the following question arises: How does the evolving socio-cultural milieu impact the interplay between anticipated instrumental support, particularly from varied sources, and depression in older adults? Thirdly, while children play a significant caregiving role, spouses are equally pivotal in older adults’ lives (37). Hence, exploring the influence of anticipated instrumental support from spouses on depression warrants deeper exploration. In addressing these gaps, this study harnesses the 2018 CHARLS dataset, encompassing both intergenerational support and anticipated instrumental support metrics, to analyze their effects on depression in older adults. The fact that the nature of support (anticipated, provided, or received) and its source (spouses, children, or others) could have intertwined effects on depression must be recognized. By concurrently assessing diverse types of received support and the different sources of anticipated instrumental support, we aim to unveil the intricate interplay of these variables. In doing so, we hope to offer a comprehensive view of their collective influence on the mental health of older adults, thereby laying the groundwork for future interventions targeting depression.

## Methods

### Data sources

This study utilized data from the 2018 wave of CHARLS, an expansive interdisciplinary survey project overseen by the National School of Development at Peking University. In 2018, CHARLS distributed questionnaires to 450 communities across 150 counties in 28 provinces, including autonomous regions and municipalities directly governed by the central government. The sampling procedure was rooted in a stratified random method. The survey encompassed 19,816 individuals aged 45 and above, of whom 10,997 were 60 or older. A total of 7,117 samples with adult children aged 60 years and older with complete records (no missing data) were included in this study, excluding those with “do not know” and “refused to answer” response options.

### Variable selection

#### Dependent variable

The outcome of interest was the presence or absence of depressive symptoms in respondents. This was gauged using the short-form Center for Epidemiologic Studies Depression Scale (CES-D-10) featured in the CHARLS questionnaire, with comparable predictive accuracy compared with the full-length 20-item CES-D (38, 39). The CES-D-10 includes 10 items, each with four graded response options. These options are scored from 0 to 3, progressing from positive to negative sentiments. The total score is derived by summing the scores from all 10 items. Following an established cutoff of 10 indicating depressive symptoms, respondents scoring 10 or above were classified as having depressive symptoms (coded as 1), while scores below 10 indicated an absence of depressive symptoms (coded as 0) (38).

#### Independent variables

Table 1 delineates the specific categories and distinctions within intergenerational support and anticipated instrumental support. Given the patterns of support provision and receipt observed among respondents, this study parsed intergenerational financial and instrumental support into four categories: non-exchange—respondents neither provided nor received any support over the past year; providing only—respondents solely provided support without receiving any in return during the preceding year; receiving only—respondents solely received support and did not offer any within the same timeframe; mutual support—respondents both provided and received support during the past year. Considering the intrinsic reciprocal quality of emotional support, it was categorized simply as either non-exchange or mutual emotional support. The expectation around future instrumental support was bifurcated into two broad categories: those who did and those who did not anticipate receiving instrumental support. Within this context, the expected sources of instrumental support were grouped into four segments: spouse and children—respondents anticipate receiving support from both these sources; spouse only—support is expected solely from the spouse; children only—support is anticipated exclusively from children; others—respondents expect support from sources other than spouses or children.

**Table 1:** Questionnaire of intergenerational support and anticipated instrumental support.

Scale	Items	Response options	Categorization
Intergenerational financial support	(1) During last year, what was the amount of Financial support provided to [Child Name]?	>0	Provide
		=0	No provide
	(2) During last year, what was the amount of Financial support received from [Child Name]?	>0	Receive
		=0	No receive
Intergenerational instrumental support	(1) During last year, did you/your spouse spend time in taking care of your grandchildren?	Yes	Provide
		No	No provide
	(2) Who most often helps you with (dressing, bathing, eating, getting out of bed, using the toilet, controlling urination and defecation, doing chores, preparing hot meals, shopping, managing money, making phone calls, taking medications)?	Children	Receive
		Other options	No receive
	(3) Do you live with your children?	Yes	Receive
		No	No receive
Intergenerational emotional support	(1) When [ChildName] is not living with you, How often do you see each other?	≥1 time per week	Receive
		<1 time per week	No receive
	(2) When [ChildName] is not living with you, How often do you contact with [Child Name] on phone/by message/on WeChat/by mail/by email?	≥1 time per week	Receive
		<1 time per week	No receive
Anticipated instrumental support	(1) Suppose that in the future, you needed help with basic daily activities like eating or dressing. Do you have relatives or friends (besides your spouse/partner) who would be willing and able to help you over a long period of time?	Yes	Available
		No	Not available
	(2) What is the relationship to you of that person or those persons?	Spouse and children	Spouse and children
		Spouse	Spouse only
		Children	Children only
		Other options	Others

#### Control variables

Existing literature has emphasized that depression in older adults is modulated by many individual factors (40–42). To account for these multifaceted influences, this study incorporated several control variables, including gender, age, marital status, place of residence, education level, health insurance, and self-rated health. Marital status was segmented into two primary categories: (1) married—this encompassed individuals who were married and cohabitating with their spouse and those married but residing separately because of work-related reasons; (2) unmarried—this broader category included individuals who were separated (not cohabitating with their spouse), divorced, widowed, or had never married. The specific classifications and corresponding details for each variable can be found in Table 2.

**Table 2:** Variables and assignments.

	Variable	Assignment
Dependent variable	Depressive symptoms	0 = no depressive symptoms; 1 = with depressive symptoms
Independent variables	Intergenerational financial support	0 = no exchange; 1 = only provide; 2 = only receive3 = mutual support
	Intergenerational instrumental support	0 = no exchange; 1 = only provide; 2 = only receive3 = mutual support
	Intergenerational emotional support	0 = no exchange; 1 = mutual support
	Anticipated instrumental support	0 = not available; 1 = spouse and children; 2 = spouse only; 3 = children only; 4 = others
Control variables	Gender	0 = male; 1 = female
	Age	0 = 60–64 years; 1 = 65–69 years; 2 = 70–74 years; 3 = over 75 years
	Marital status	0 = unmarried; 1 = married
	Residence	0 = urban; 1 = rural
	Education	0 = illiteracy; 1 = primary school; 2 = junior high school; 3 = senior high school and above
	Type of medical insurance	0 = no insurance; 1 = medical insurance for urban workers; 2 = medical insurance for urban residents; 3 = new rural cooperative medical insurance
	Self-rated health status	0 = excellent; 1 = good; 2 = fair; 3 = poor; 4 = very poor

## Results

### Demographic overview of the respondents

Of the 7,117 respondents considered in this study, 3,513 (49.36%) and 3,604 (50.64%) identified as male and female, respectively. A significant majority, 5,782 (81.24%), were in marital unions, and 1,335 (18.76%) were not. Regarding place of residence, 5,219 (73.33%) resided in rural settings, with the remaining 1,898 (26.67%) living in urban areas. Given China’s varied gender and regional dynamics, which include distinct urban and rural contexts, our sample’s balanced representation from these groups suggests that it effectively mirrors the wider older Chinese population. Notably, 38.89% of the older respondents exhibited depressive symptoms, and an encouraging 71.13% anticipated the availability of instrumental support. Detailed respondent demographics are available in Table 3.

**Table 3:** Basic characteristics of survey samples (*n* = 7,117, n/%).

Category	*n*	%	Category	*n*	%
Gender			Self-rated health status		
Male	3,513	49.36	Very good	777	10.92
Female	3,604	50.64	Good	802	11.27
Age			Fair	3,473	48.80
60–64 years	2051	28.82	Poor	1,597	22.44
65–69 years	2,237	31.43	Very poor	468	6.58
70–74 years	1,435	20.16	Intergenerational financial support		
Over 75 years	1,394	19.59	No exchange	1,172	16.47
Marital status			Only provide	395	5.55
Unmarried	1,335	18.76	Only receive	3,778	53.08
Married	5,782	81.24	Mutual support	1772	24.90
Residence			Intergenerational instrumental support		
Urban	1898	26.67	No exchange	2,356	33.10
Rural	5,219	73.33	Only provide	1,530	21.50
Type of medical insurance			Only receive	1813	25.47
No insurance	359	5.04	Mutual support	1,418	19.92
Medical insurance for urban workers	1,036	14.56	Intergenerational emotional support		
			No exchange	1,298	18.24
Medical insurance for urban residents	1,205	16.93	Mutual support	5,819	81.76
			Anticipated instrumental support		
New rural cooperative medical insurance	4,517	63.47	Not available	2055	28.87
			Spouse and children	1,534	21.55
Education			Spouse only	784	11.02
Illiteracy	1865	26.20	Children only	2,650	37.23
Primary school	3,282	46.11	Others	94	1.32
Junior high school	1,215	17.07	Depressive symptoms		
Senior high school and above	755	10.61	Yes	4,349	61.11
			No	2,768	38.89

### Determinants of depression in older adults

Table 4 presents the correlation analysis between individual factors and the manifestation of depressive symptoms among respondents. The findings revealed that, except for age, all control variables were significantly associated with depressive symptoms. Further, every independent variable—encompassing intergenerational financial, instrumental, and emotional support, and anticipated instrumental support—displayed a significant correlation with depressive symptoms in this age group.

**Table 4:** Correlation analysis of the influencing factors of depressive symptoms (*n* = 7,117, n/%).

Category	Non-depressive	Depressive	Chi-sq	*p*-value
*Gender*				
Male	2,414(55.51)	1,099(39.70)	169.00	0.00
Female	1935(44.49)	1,669(60.30)		
*Age*				
60–64 years	1,261(28.99)	790(28.54)	2.76	0.43
65–69 years	1,383(31.80)	854(30.85)		
70–74 years	850(19.54)	585(21.13)		
Over 75 years	855(19.66)	539(19.47)		
*Marital status*				
Married	3,644(83.79)	2,138(77.24)	47.62	0.00
Unmarried	705(16.21)	630(22.76)		
*Residence*				
Urban	1,311(30.14)	587(21.21)	69.10	0.00
Rural	3,038(69.86)	2,181(78.79)		
*Education*				
Illiteracy	983(22.60)	882(31.86)	176.54	0.00
Primary school	1938(44.56)	1,344(48.55)		
Junior high school	851(19.57)	364(13.15)		
Senior high school and above	577(13.27)	178(6.43)		
*Self-rated health status*				
Very good	647(14.88)	130(4.70)	813.80	0.00
Good	625(14.37)	177(6.39)		
Fair	2,303(52.95)	1,170(42.27)		
Poor	652(14.99)	945(34.14)		
Very poor	122(2.81)	346(12.50)		
*Type of medical insurance*				
No insurance	224(5.15)	135(4.88)	133.60	0.00
Medical insurance for urban workers	788(18.11)	248(8.96)		
Medical insurance for urban residents	765(17.59)	440(15.90)		
New rural cooperative medical insurance	2,572(59.14)	1945(70.29)		
*Intergenerational financial support*				
No exchange	647(14.88)	525(18.97)	63.65	0.00
Only provide	231(5.31)	164(15.92)		
Only receive	2,255(51.85)	1,523(55.02)		
Mutual support	1,216(27.96)	556(20.09)		
*Intergenerational instrumental support*				
No exchange	1,388(31.92)	968(34.97)	11.14	0.01
Only provide	983(22.60)	547(19.76)		
Only receive	1,109(25.50)	704(25.43)		
Mutual support	869(19.98)	549(19.83)		
*Intergenerational emotional support*				
No exchange	697(16.03)	601(21.71)	36.67	0.00
Mutual support	3,652(83.97)	2,167(78.29)		
*Anticipated instrumental support*				
Not available	1,030(23.68)	1,025(37.03)	180.10	0.00
Spouse and children	1,089(25.04)	445(16.08)		
Spouse only	466(10.72)	318(11.49)		
Children only	1702(39.14)	948(34.25)		
Others	62(1.43)	32(1.16)		

### Evaluating the influence of intergenerational support and anticipated instrumental support on depression among older adults

**Figure 1:**
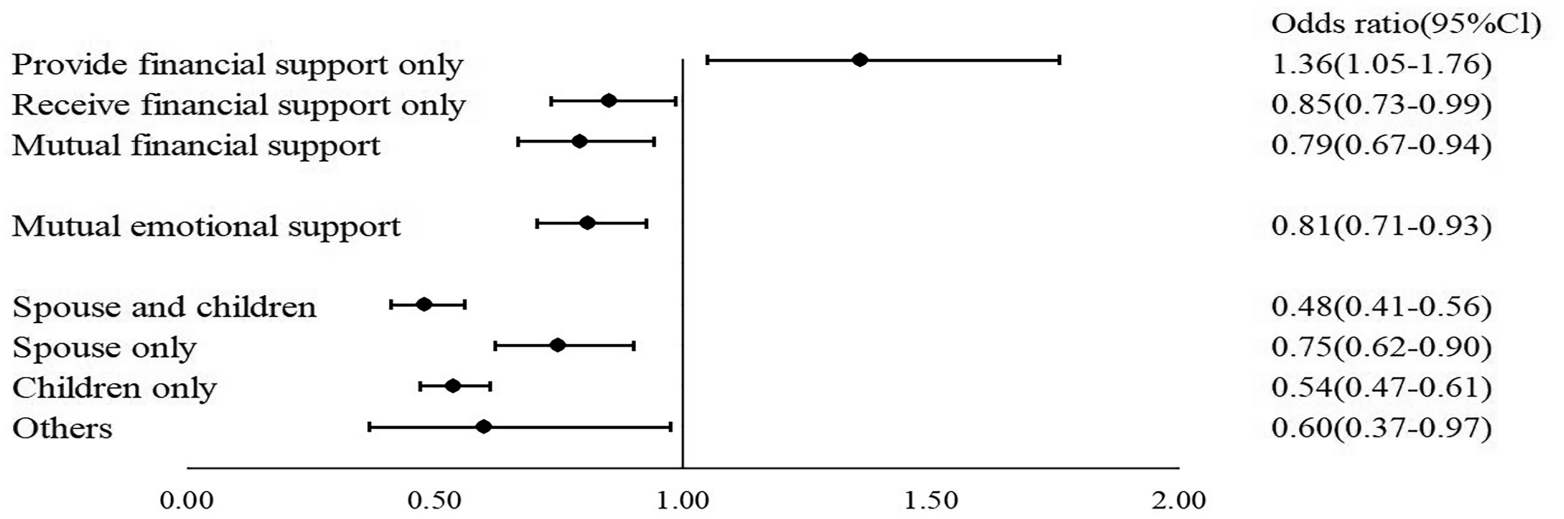
Forest diagram of factors influencing depressive symptoms in older adults.

This study employed three binary logistic regression models, using the occurrence of depressive symptoms among respondents as the dependent variable. In model 1, control variables that exhibited significant correlations with the manifestation of depressive symptoms in the univariate analysis were included to assess their influence on depression. Building upon model 1, model 2 integrated the three domains of intergenerational financial, instrumental, and emotional support to examine their collective impact on depression in this demographic. Model 3, an extension of model 2, introduced the variable of anticipated instrumental support to examine its influence according to different anticipated sources. The Hosmer test results for all models exceeded 0.05, indicating a satisfactory model fit (43). For a detailed breakdown, refer to Table 5 and Figure 1.

**Table 5:** Regression analysis of the effects of anticipated instrumental support on depressive symptoms in older adults (*n* = 7,117).

Category	Model 1	Model 2	Model 3
Gender (male = 0)			
Female	1.71(1.53–1.92)^***^	1.75(1.57–1.96)^***^	1.78(1.59–2.00)^***^
Marital status (unmarried = 0)			
Married	0.78(0.68–0.89)^***^	0.79(0.69–0.90)^***^	0.80(0.69–0.92)^**^
Residence (urban = 0)			
Rural	1.13(0.98–1.30)	1.12(0.97–1.29)	1.14(0.98–1.32)
Education (illiteracy = 0)			
Primary school	1.01(0.89–1.15)	1.01(0.89–1.16)	1.03(0.90–1.18)
Junior high school	0.81(0.68–0.96)^*^	0.82(0.68–0.98)^*^	0.82(0.68–0.98)^*^
Senior high school and above	0.68(0.54–0.85)^**^	0.69(0.55–0.86)^**^	0.68(0.54–0.85)^**^
Type of medical insurance (no insurance = 0)			
Medical insurance for urban workers	0.65(0.49–0.86)^**^	0.64(0.48–0.85)^**^	0.66(0.49–0.88)^**^
Medical insurance for urban residents	0.87(0.67–1.13)	0.87(0.67–1.14)	0.93(0.71–1.21)
New rural cooperative medical insurance	1.07(0.84–1.37)	1.08(0.84–1.38)	1.15(0.89–1.47)
Self-rated health status (very good = 0)			
Good	1.47(1.14–1.90)^**^	1.46(1.13–1.89)^**^	1.49(1.05–1.76)^**^
Fair	2.55(2.08–3.12)^***^	2.55(2.08–3.13)^***^	2.53(2.06–3.11)^***^
Poor	6.76(5.44–8.39)^***^	6.74(5.43–8.38)^***^	6.58(5.28–8.19)^***^
Very poor	14.06(10.58–18.69)^***^	14.17(10.65–18.86)^***^	13.57(13.57–18.12)^***^
Intergenerational financial support (no exchange = 0)			
Only provide		1.36(1.05–1.75)^*^	1.36(1.05–1.76)^*^
Only receive		0.81(0.70–0.94)^**^	0.85(0.73–0.99)^*^
Mutual support		0.77(0.65–0.91)^**^	0.79(0.67–0.94)^**^
Intergenerational instrumental support (no exchange = 0)			
Only provide		0.88(0.76–1.02)	0.90(0.78–1.05)
Only receive		0.86(0.75–0.99)^*^	0.89(0.78–1.02)
Mutual support		0.93(0.80–1.07)	0.98(0.84–1.14)
Intergenerational emotional support (no exchange = 0)			
Mutual support		0.76(0.67–0.87)^***^	0.81(0.71–0.93)^**^
Anticipated instrumental support (not available)			
Spouse and children			0.48(0.41–0.56)^***^
Spouse only			0.75(0.62–0.90)^**^
Children only			0.54(0.47–0.61)^***^
Others			0.60(0.37–0.97)^*^

*<0.05; **<0.01; ***<0.001.

Model 1 evaluated the influence of control variables on depressive symptoms among the older population. The findings were as follows: (1) Women had 1.71 times the odds of experiencing depressive symptoms compared with men. (2) Married individuals had 22% lower odds of manifesting depressive symptoms than their unmarried counterparts. (3) Compared with those without formal education, individuals with junior high school education and high school or higher education had 19 and 32% reduced odds of showing depressive symptoms, respectively. (4) Older adults covered by urban employee health insurance had 35% reduced odds of experiencing depressive symptoms relative to those without any health insurance. (5) Contrary to individuals who rated their health as “very good,” the odds of having depressive symptoms for those whose self-rated health was “good,” “fair,” “poor,” and “very poor” were 1.47, 2.55, 6.76, and 14.06 times higher, respectively. (6) No significant differences in the odds of depressive symptoms were observed between urban and rural dwellers, those with primary education and those without formal education, or those without insurance and those with different health insurance types, such as urban residents’ health insurance and new rural cooperative insurance.

Model 2 assessed the influence of intergenerational support on depressive symptoms among the older population. The findings were as follows: (1) Regarding intergenerational financial support, older adults who solely provided financial support had 1.36 times the odds of experiencing depressive symptoms compared with those not engaged in any financial exchange; Those who only received and those engaged in mutual financial support had 19 and 23% reduced odds of depressive symptoms, respectively, compared with those without any financial exchange. (2) For intergenerational instrumental support, older adults who solely received instrumental support had 23% reduced odds of experiencing depressive symptoms compared with those not involved in any instrumental exchange. No significant difference in the odds of depressive symptoms was observed between individuals only providing instrumental support, those with mutual instrumental support, and those without any instrumental exchange. (3) For intergenerational emotional support, older adults with mutual emotional support had 24% reduced odds of manifesting depressive symptoms compared with those without any emotional exchange.

Finally, Model 3 explored the influence of various sources of anticipated instrumental support on depressive symptoms in the older population. The analysis revealed that anticipating support from different sources acted as protective factors against depression. Specifically, older adults who anticipated spousal and child support, spousal support only, child support only, or support from other sources had 52, 25, 46, and 40% reduced odds of experiencing depressive symptoms, respectively, compared with those without such expectations.

## Discussion

### The role of anticipated instrumental support in protecting older adults from depression

After adjusting for the effects of intergenerational support and individual-related factors, older adults without an anticipation of receiving instrumental support displayed notably higher odds of developing depressive symptoms. The odds of depressive symptoms were reduced by 52, 25, 46, and 40% for those anticipated instrumental support from spouse and children, spouse only, children only, and other sources, respectively, compared with their counterparts without such anticipation. These findings align with those of Cheng (29), who used data from the 2011 and 2013 waves of CHARLS (29).

The current study delved deeper into the effects of different sources of anticipated instrumental support—spouse and children, spouse only, children only, and other sources—on depressive symptoms in older adults. The results showed that all support sources significantly reduced the odds of depression, with varying magnitudes. We found that anticipated support from spouses and children exhibited the most significant protective effect against depression. These outcomes emphasize the evolving role of anticipated instrumental support in safeguarding older adults against depression as societal dynamics shift. Such effects can be traced back to traditional Chinese cultural values and the prevalent social welfare system. In China, caregiving within the household remains paramount, with spouses and children being the primary caregivers for older adults (44). The anticipation of receiving instrumental support, particularly from family members, lessens anxieties among older adults.

However, recent demographic shifts have seen the family living structure in China transition from predominantly multi-generational households to more nuclear configurations. The number of older adults living solely with their spouses or alone has increased (45). Further, the anticipation of receiving instrumental support from children has been declining (29). This evolution underscores the burgeoning importance of anticipated support and its heightened influence on the mental well-being of older adults. Reliable and trusted caregiving anticipation, especially from close family members, can alleviate mental stressors. The absence of this anticipation can amplify anxieties and the potential for depression. Notably, the anticipation of support from other individuals appeared to reduce depressive symptoms more effectively than from a spouse alone (40% vs. 25%). This might be attributed to the fact that spouses are typically of a similar age, which introduces uncertainty regarding their caregiving capabilities and potentially intensifies stress. Compared with spousal support, the anticipation of assistance from other sources, possibly younger or more capable individuals, offers a more certain relief, reducing the caregiving burden on families and mitigating feelings of guilt in the older generation (27).

### Impact of various patterns of intergenerational support on depressive symptoms in older adults

#### Financial support

Older adults who solely provided financial assistance to their younger generations were found to be more susceptible to depression. Given that a considerable number of respondents (73.33%) resided in rural areas with relatively lower income levels, financially supporting their children could compromise their quality of life, which can, in turn, impact their overall well-being. This observation aligns with the findings of Zhang et al. (46). On the contrary, older adults who were solely on the receiving end or engaged in mutual financial transactions with their children had a reduced risk for depression. Such financial contributions from children can bolster a sense of accomplishment in older adults and alleviate financial stressors, further mitigating depressive tendencies. Importantly, mutual financial support demonstrated a more substantial protective effect against depression than merely receiving support, reducing depressive tendencies by 19% vs. 23%. Older adults providing support to children often reflects a sound financial foundation, while receiving support can be interpreted as a gesture of gratitude by the children. Such mutual financial support indicates solid intergenerational bonds and plays a pivotal role in warding off depression (47).

#### Instrumental support

Our findings indicated that older adults who exclusively received instrumental support showcased reduced depressive symptoms. Such support not only streamlines the daily undertakings of older adults but also provides more opportunities for them to bond with their children, which is instrumental in alleviating their anxiety.

#### Emotional support

Mutual emotional exchanges were identified as a protective factor against depression, a finding that resonates with Choi et al. (48). Strengthened emotional bonds are believed to intensify the connection between older adults and their offspring, minimizing the risk associated with depressive episodes (17).

### Multiple factors influencing depressive symptoms in older adults

This study showed that various factors, such as gender, marital status, education level, health insurance, and self-rated health, significantly correlate with depressive symptoms among older adults. Within the sample of this study, 60.30% of older women exhibited depressive symptoms, a figure notably higher than that of their male counterparts. This suggests that older women face a greater susceptibility to depression compared with older men. Marital status emerged as another critical determinant. Relative to their married peers, those who remained unmarried often displayed signs indicative of diminished emotional comfort during their later years. Given the persistence of feelings such as loneliness, the onset of depressive symptoms becomes more probable (49). Further, a positive correlation was observed between superior self-rated health and reduced depressive symptoms. This self-assessment is not merely a reflection of their physical state but also encompasses their perception of their health, which can, in turn, mirror their depressive state (50, 51).

This study comprehensively explored the effects of anticipated instrumental support, diverse types of intergenerational backing, and individual determinants on depression in older adults, offering a more nuanced understanding of the causative factors. However, certain limitations must be acknowledged. First, the utilization of cross-sectional data only permitted an analysis of the contemporary effects of the variables on depression without considering dynamic shifts over time. Future research employing panel data could delve deeper into these evolving dynamics. Second, this study did not factor in potential overlaps and synergies between the diverse intergenerational support and anticipated instrumental support. Because anticipated instrumental support may be influenced by intergenerational exchange, including content, direction, and recency (25). Additionally, the impact of different levels of anticipated instrumental support and intergenerational support on depression in older adults was not considered in this study. Prior research has found that older adults with ‘high receipt and low expectations’ were associated with fewer depressive symptoms, while older adults with “high expectations and low receipt” were associated with greater depressive symptoms, which might introduce some bias to the findings (28). In the future, we can employ structural equation models to explore the effects of different levels and sources of anticipated instrumental and intergenerational support on depressive symptoms in older adults.

Despite the limitations, our paper found that different sources of anticipated instrumental support and intergenerational support have significant effects on depressive symptoms in older adults and have important theoretical and policy implications. Firstly, our study offers new insights into research on depressive symptoms in older adults by combining the analysis of different sources of anticipated instrumental support and intergenerational support. Previous studies have predominantly focused on the availability of anticipated instrumental support, overlooking the distinct impact of various sources on depressive symptoms in older adults. Secondly, our findings can serve as a valuable reference for state policymakers in formulating relevant policies. Different sources of anticipated instrumental support represent the assessments and expectations of older adults in hypothetical situations. These insights can help us better understand the social and familial needs of older adults, providing essential guidance for the development of future intervention measures. Against the backdrop of an increasingly aging population and a consistently low fertility rate, the healthcare and long-term care requirements of older adults have increased. Moreover, the absence of a robust social security system, coupled with the early stage of the long-term care insurance system, intimately associated with older adults, has intensified their dependence on anticipated instrumental support from their offspring. To attenuate depressive symptoms, especially those resulting from an anticipated lack of instrumental support, policymakers must holistically address care provisions for older adults and reduce the caregiving burden on families. Furthermore, fostering a more pronounced caregiving responsibility ethos within families, particularly among spouses and offspring, can enhance the resilience of older adults against potential future challenges.

## Data availability statement

The original contributions presented in the study are included in the article/supplementary material, further inquiries can be directed to the corresponding authors.

## Ethics statement

All the respondents signed informed consent at the time of participation, and this study was approved by the Institutional Review Board of Peking University (IRB00001052–11014). The studies were conducted in accordance with the local legislation and institutional requirements. Written informed consent for participation in this study was provided by the participants’ legal guardians/next of kin.

## Author contributions

DF: Writing – original draft, Writing – review & editing. FW: Writing – review & editing. BG: Writing – review & editing. QB: Writing – review & editing. GL: Writing – review & editing, Funding acquisition. JZ: Writing – review & editing.
